# Nanoparticle Functionalization and Its Potentials for Molecular Imaging

**DOI:** 10.1002/advs.201600279

**Published:** 2016-12-16

**Authors:** Rukmani Thiruppathi, Sachin Mishra, Mathangi Ganapathy, Parasuraman Padmanabhan, Balázs Gulyás

**Affiliations:** ^1^ Lee Kong Chian School of MedicineNanyang Technological University59 Nanyang Drive636921Singapore; ^2^ Center for BiotechnologyAlagappa College of TechnologyAnna UniversitySardar Patel RoadChennaiTamil Nadu600025India

**Keywords:** conjugation, functionalization, imaging, microscopy, nanoparticles

## Abstract

Functionalization enhances the properties and characteristics of nanoparticles through surface modification, and enables them to play a major role in the field of medicine. In molecular imaging, quality functional images are required with proper differentiation which can be seen with high contrast to obtain viable information. This review article discusses how functionalization enhances molecular imaging and enables multimodal imaging by which images with combination of functions particular to each modality can be obtained. This also explains how nanoparticles interacting at molecular level, when functionalized with molecules can target the cells of interest or substances with high specificity, reducing background signal and allowing simultaneous therapies to be carried out while imaging. Functionalization allows imaging for a prolonged period and enables to track the cells over a period of time. Recent researches and progress in functionalizing the nanoparticles to specifically enhance bioimaging with different modalities and their applications are reviewed in this article.

## 1 Introduction

Nanoparticles (NPs) owing to their size have many distinct and unique native properties. The nanoscale size allows these NPs to have interaction at the molecular level. There are several noninvasive techniques used for molecular imaging, and there exists numerous ways through which they can be improved for a better quality imaging.^1^ Multifunctional NPs are known to enhance the way of monitoring and revealing molecular level events. Enhancing the imaging through NP‐based probes, like creating high contrast images, is the most efficient way to get improved results.^2^ Therefore, greater concentration toward nanoprobe development is essential in present research scenario.

The applications of NPs were considered to be very limited due to their low targeting efficiency and high toxicity levels.^3^, ^4^ The unprecedented incorporation of NPs for imaging can even cause irreversible molecular and cellular damage. In the absence of any molecular moiety being attached, these NPs generally show nonselective distribution across the body and inability to overcome the biological hindrance like Blood Brain Barrier (BBB), and thus, fail to satisfy the preconditions necessary for molecular imaging. Their surface properties have to be modified for use in various fields of enhanced imaging and to overcome the little drawbacks these nanoscale particles had earlier.

The era of nanoprobes development paved the way for research on functionalization of NPs to aid various approaches of imaging techniques. Ultimately to diagnose the disease and to monitor the delivery and effect of these NPs, we need efficient molecular imaging methods. Functionalized nanoprobes will serve the multidimensional purpose as a few NPs can not only interact at the molecular level, but also have superior optical properties which sometimes can be tuned with respect to the size, like in the case of carbon nanodots.^5^ Also for the probes to be compatible with the advancements in the instrumentation of imaging modality, like in case of hybrid imaging, functionalization is required.

Functionalization refers to the surface modification of NPs, which includes conjugation of chemicals^6^ or bio molecules on to the surface like folic acid, biotin molecules, oligo nucleotides, peptides, antibodies, etc., to enhance the properties and hit the target with high precision.^7^ In addition to this, the functionalized NPs have good physical properties, anti‐corrosion, anti‐agglomeration and noninvasive characteristics. Intense researches has been carried out to functionalize the NPs to enhance its overall efficiency and modality.^8^, ^9^

Functionalization of NPs allows us to inculcate properties that we are specifically interested to incorporate in NPs, thus, it can be done to aid a specific imaging modality approach. It not only helps in getting better image quality, but also increases the applicability and functionality of the imaging modality. In various researches, functionalization of NPs is also found to be done with another NP for enhancing its characteristics. Qu et al. worked with silica NP which have high biocompatibility, was layered with magnetite NPs, and further functionalized with dyes on the magnetite layer.^10^ The silica NPs which cannot be used as contrast agents (CAs) earlier can be utilized as T2‐weighted CAs in magnetic resonance imaging (MRI) and also in optical imaging. This shows that there exists a dependency of imaging techniques on functionalization approaches. The reliability on the images depends on the specificity of the probes to avoid false positives, and therefore, to develop highly reliable imaging techniques for diagnostic purposes, ideal probe development strategies are needed. Since only after diagnosis, one can take important decisions on the type of disease and the intensity of treatment, apt probes with suitable surface properties is needed.

This review paper discusses the interdependency of NP functionalization and molecular imaging techniques, which aids the imaging approaches in functionality and applicability. The most recent researches available in this field from the year 2012 to 2016 are reviewed. The chemistry behind these functionalization approaches including noncovalent binding, covalent conjugation, surface coating and others are discussed. An overview is provided how functionalization can enhance and assist specific imaging techniques like confocal microscopy, fluorescence imaging, MRI, positron emission tomography (PET), and computed tomography (CT), for in vitro and in vivo studies. Functionalization enables loading NPs with multimodal characteristics, targeted functionalities and possibilities of various intraoperative procedures to be carried out. The various other domains related to molecular imaging which have benefited from functionalization are also discussed, such as theranostics, multimodal and hybrid imaging, intraoperative therapies and molecular targeting.

## 2 Chemistry of Functionalization

**Figure 1:**
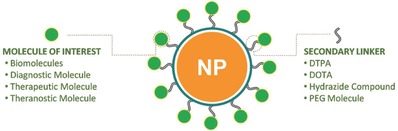
Nanoparticles can be linked to various diagnostic and therapeutic molecules of interest through secondary linkers when conjugation is not possible through on‐surface reactive groups.

The chemistry of functionalization determines the interaction of nanoprobe with biological environment and also with the imaging modality technique. Various approaches of functionalization of NPs are explored as per the applicability, efficiency and other requirements of molecular imaging. **Figure** **1** provides an overview of molecules of interest which can be attached to the NPs through secondary linkers. Functionalization of NPs involves conjugation of molecules on the surface of the particles. The high surface to volume ratio allows efficient functionalization of particles to suit our needs. It is important to know about the chemistry behind the conjugation as it would enable us to know the feasibility of various functionalization approaches.

### 2.1 Noncovalent Binding

Conjugation can happen through noncovalent interactions by attaching some specific secondary ligands. Noncovalent conjugation of biomolecules on NPs is generally known to be performed through affinity‐based receptor‐ligand systems. Various approaches of noncovalent binding include electrostatic interaction, π–π stacking and entrapping biomolecules in biocompatible films like phospholipids, polymer, DNA, etc.^11^ Agudelo and co‐workers showed that tRNA molecules can be noncovalently attached to the chitosan NPs through H‐bonding and electrostatic interactions via G–C and A–U base pairs.^12^ Li et al. worked with cadmium telluride (CdTe) quantum dots (QDs) for luminescent sensing of lysozyme by conjugating it with lysozyme binding DNA, through electrostatic interactions.^13^

### 2.2 Covalent Conjugation

Functionalization is also possible by direct binding of molecule of interest to the reactive ligands on NP surface, facilitated by covalent conjugation. This approach involves linkage reaction aided by catalyst and is mostly preferred with unspecific physisorption in terms of stability of functionalization.^11^ Svenson et al. proved that, instead of electrostatic attraction, covalent linkers can be used to form poly(d,l‐lactic‐*co*‐glycolic acid) (PLGA)—Small interfering RNA (siRNA) conjugates for efficient release of siRNA molecules.^14^ Various approaches of covalent conjugation include chemisorption via thiol derivatives, bifunctional linkers/mediator linkers, adapter molecules such as streptavidin and biotin and dative binding.

### 2.3 Amorphous Nanoparticle Coating

Inorganic NPs like metal NPs have to be coated with silica or some other polymers which can introduce functional groups to them.^15^ Surface coating also imparts additional functionality to the NP as it allows targeting ligand/therapeutic attachment. Konduru et al. coated amorphous silica on zinc oxide NPs to alter its biokinetic behavior and biological responses in rats by affecting protein corona, agglomerate size and zeta potential.^16^ NPs can be coated with organic (monomer and polymer) and inorganic (metal and oxides) layers. Among the metals, gold and silica are well explored to be coated on NPs to induce high stability and conductive environment for subsequent functionalization. As coating may hinder the magnetic/physical properties of NPs, it should be taken in consideration for imaging applications.^17^

### 2.4 Surface Epitaxial Growth

Epitaxial approach involves modification of NP surface by depositing an inorganic or organic overlayer on a substrate. Bifunctional composite NPs can be obtained by epitaxial growth of iron oxide NPs (IONPs) on the Au seeds to get Au−Fe_3_O_4_ NPs, by decomposing Fe(CO)_5_ on AuNP surface followed by oxidation.^18^ Hachtel et al. worked synthesized microwave assisted gold nanotriangles decorated with IONPs, providing both magnetic and plasmonic properties to the particle.^19^ Epitaxial approach provides a wider scope of multifunctional nanoprobes development.

The mentioned approaches and chemistry of functionalization, together with various other methods, provides a wide scope of functionalizing NPs to enhance it for molecular applications and other biological applications.

## 3 Functionalization to Enhance Biocompatibility

Surface modification of NPs affects various characteristics including size, surface chemistry, hydrophilicity and hydrophobicity, charge, etc., thus could alter the solubility, biocompatibility, biodistribution, and clearance of developed nanoprobes.

NPs when coated with small molecule or polymer like dextran, starch, citrate or poly(ethylene glycol) (PEG), the developed nanoprobes shows enhanced biodistribution with longer circulation time and efficient uptake. Hydrophilic polymers, like PEG, can be conjugated with NPs to provide steric stabilization and thus preventing protein absorption.^20^ Also, it exhibits prolonged blood circulation for in vivo administration. Modified surface chemistry of NPs can significantly affect their in vivo pharmacokinetic behavior. Linking superparamagnetic iron oxide NPs (SPIONs) with PEG molecules is known to increase the hydrophilicity, stability and to reduce cytotoxicity levels.^21^ Ulusoy et al. showed that QDs CdTe/CdS/ZnS heavily functionalized with mPEG (methoxy PEG) showed a severe decrease in the deposition of the ZnS, increase in the colloidal stability suitable to cure solid tumors and also improved binding affinity with decrease in fluorescence only by 3%.^22^ Due to increased binding affinity, cellular interactions were clearly visible through confocal microscopy.

Clearance of NPs is an important aspect for its applicability in molecular imaging, and renal excretion represents a desirable pathway with minimal catabolism. Functionalization can alter physical properties of NPs and can make it effective for clearance. Clearance studies with polyamidoamine dendrimer‐based NPs shows effective glomerular filtration, equivalent to conventional molecules.^23^ Choi et al. worked with QDs made of CdSe core and a ZnS shell, coated with anionic, cationic, zwitterionic or neutral small molecules.^24^ The result showed rapid and efficient urinary excretion of QDs for zwitterionic or neutral organic coatings as it prevented adsorption of serum proteins.

It can be conferred that surface modification of NPs induces biocompatible characteristics to nanoprobes, which is essential for proper administration, targeting, biodistribution and clearance in molecular imaging applications.

## 4 Functionalization to Enhance Imaging

Various approaches of functionalization of NPs are explored by researchers to specifically enhance the molecular images acquired with different modalities, both in vitro and in vivo. These functionalized nanoprobes are capable of interacting in accordance with the imaging technique, and thus revealing the desired structural and/or functional information.

### 4.1 Confocal Microscopy

**Figure 2:**
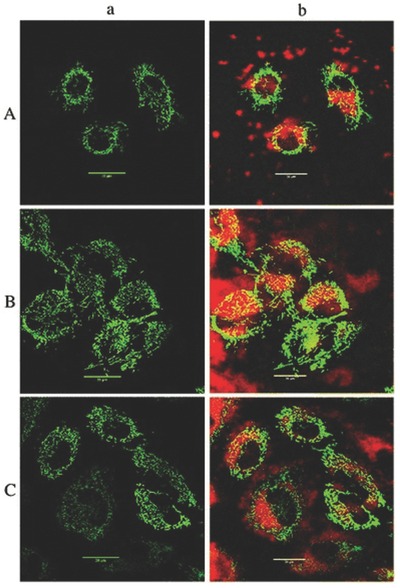
a) Confocal fluorescence microscopy images of mitochondria at 480 nm in mRoGFP HeLa cells after incubation with hexagonal A) NaYF_4_:Yb^3+^/Er^3+^, B) NaYF_4_:Yb^3+^/Er^3+^&SiO_2_, and C) NaYF_4_:Yb^3+^/Er^3+^&SiO_2_‐NH_2_ nanocrystals. b) Overlays of (a) and upconversion luminescence (excitation at 970 nm and detection at 500–700 nm). Adapted with permission.^25^ Copyright 2015, The Royal Society of Chemistry.

In vitro analysis allows us to see how successful an experiment is, through simple means. Confocal microscopy allows imaging the cell culture at different planes, and recombining them provides a 3D image. It helps to negate the auto fluorescence by making use of the different excitable states existing in the biomolecules and the fluorescent dye. The drug delivery mechanisms can be studied in vitro. Therefore, probes which could enhance the images obtained through confocal microscopy are constantly being developed. Nanoprobes are also used for in vitro cell uptake analysis. NaYF4:Yb^3+/^Er^3+^ nanocrystals can be used for imaging through fluorescence microscopy. Kostiv et al. tried linking the trimethoxysilane to the silica coated NPs, then the amine groups were introduced on their surfaces to which biomolecules can be attached to target them to specified location and making cellular uptake of the NPs possible through transcytosis, which can be imaged using confocal microscopy for precise analysis as shown in **Figure** **2**, 25

Zarebkohan et al. worked with confocal microscopy and identified that the Serine‐Arginine‐Lueucine functionalized dendrimer NP can cross the BBB and deliver the gene loaded in the particles.^26^ Confocal microscopy helped to confirm the entry of NPs across the BBB in vitro using blood capillary cells lining the BBB. Passivated upconversion NPs (UCNPs) enhance the emission by preventing the energy loss and help the build‐up of energy in absorbing ions to give greater brightness. Gu et al. reported that functionalizing dyes on to the UCNPs can be used to detect the Hg^2+^ concentration through the blue shift viewed through confocal microscopy which happens because of the reaction between the dye (pure compound) and the Hg^2+^.^27^ This increases the utility of the confocal microscopy. QDs are one important category of probes which could be used in fluorescence imaging. Their use was limited due to the high cytotoxicity levels. Imaging particular regions of interest through fluorescent nanoprobes via confocal microscopy was made possible in a research conducted by Michalska et al. in linking LTVSPWY peptide to the CuInZnxS2+x QDs.^28^ The NPs made out of dendrimers with attached organic dyes have ‘soft’ fluorescence. Therefore, QDs functionalized with TSPO ligands enabled Fanizza et al. to create highly luminescent probes which can be used to visualize mitochondria through confocal laser microscopy.^29^ Ruthenium functionalized NP complexes can be enhanced selectively and also the emission properties of these particles are distance dependent. Martínez‐Calvo et al. showed that Ru(II) can bind to the DNA though groove binding which creates alterations in the photo properties. It can be captured by the confocal microscopy to analyze the induction of apoptosis in the cells because of binding DNA with Ru complexes.^30^ Tamborini et al. showed the cellular uptake of PLGA NPs functionalized with chlorotoxin which specifically targets glioma cells and reported that it can be analyzed by doping optical CAs like Ag NPs.^31^

### 4.2 Fluorescence Imaging

Fluorescence imaging can effectively image the interactions happening at the molecular level with very high sensitivity and can detect the expression levels. To better utilize the imaging system, nanoprobes can be tuned accordingly. Researches have explored various methods based and NP functionalization to enhance fluorescence imaging.

High‐resolution fluorescence microscope, together with functionalized nanoprobes are being used for imaging deep inside biological tissues. Pajović et al. reported that functionalization of gold NPs with multiple layers of tryphtophan yields particles with varying fluorescent properties as this bioconjugation increase the lifetime of excited states.^32^ Since tryptophan absorbs and emits in the UV range, deep ultraviolet imaging can be done. Multiphoton imaging (MPI) helps in reducing the signal to noise ratio since auto fluorescence is minimized as biomolecules have a low two photon cross section. Instead of QDs doped with heavy metals, Natalio et al. in their study confirmed that silica particles can be used in MPI after functionalizing with SOCl_2_ and with *N*‐Boc‐1,4‐butanediamine which introduces chlorine groups and amine groups on the surface, which further allows for conjugation of biomolecules.^33^ The SiO_2_‐N gives fluorescence characteristics to the NPs. Depth of imaging depends on the excitation light properties like transmission and focal capability. Wang et al. showed that gold nanorods functionalized with PEG are luminescent probes when excited at 1000 nm through femtosecond laser, 600 um depth of in vivo imaging of brain is done with high contrast images obtained due to three photon luminescence whereas for excitation at 760 nm, imaging is done at a depth of 430 um with two photon luminescence signals.^34^

NP specific functionalization approaches for fluorescence imaging are also explored with various types of particles including silica NPs, QDs and carbon nanostructures.

#### 4.2.1 Silica NPs

**Figure 3:**
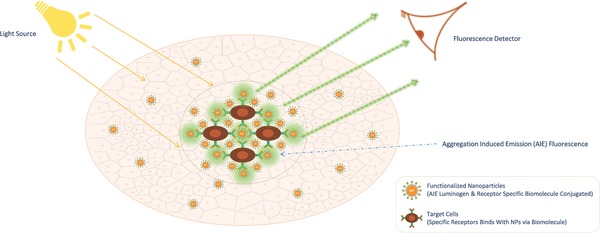
Aggregation induced emission (AIE) luminogens are fluorescent when accumulation of nanoparticles happens in tumors, which negates the false positive fluorescence and avoid confusions.

Surface of silica can be altered in several ways by linking various functional groups. Especially mesoporous particles can entrap functional groups, shielding from the external factors. Vivero‐Escoto et al. identified that when the dye tris(2,2′‐bipyridyl)dichlororuthenium(II) (Ru(bpy3))2+ complex is entrapped, higher quantum yield is obtained than the dyes alone.^35^ But leaching can reduce its efficiency therefore the use of covalent interactions to link the dye through trialkoxysilnae can be carried out. Silica particles doped with three different dyes and functionalizing the silica NPs with three different peptides or aptamers by introducing biotin linked via PEG, pave way for multi imaging simultaneously. Wang reported that silica particles when attached with aggregation induced emission (AIE) dyes through non covalent interactions, enhanced fluorescence is obtained, and further loading with the AIE luminogens will not initiate the quenching effect.^36^ Aptamer linked functionalized particles along with the AIE dyes can target the MCF 7 cells with high specificity and the aggregation of NPs in that region causes fluorescence. Through this method, we can eliminate the false positives and negate the confusions. **Figure** **3** shows the concept behind aggregation induced fluorescence. We can clearly detect the areas where tumor is accumulated and high precision intraoperative therapies can be carried out.

#### 4.2.2 Quantum Dots

QDs are used for efficient bright fluorescence imaging, but their use has become very limited to their high toxicity levels and low stability. Tasso et al. showed that controlled functionalization can happen by capping the surface with multidentate dithiol/zwitterion copolymers which serve as ligands to bind with a defined number of protein molecules which in turn can bind the functional antibodies.^37^ The zwitterion ligands attaching passivate the surface of CdSe/CdS/ZnS QDs to enhance fluorescence and also all these functionalization reduces the cytotoxicity. QDs are generally functionalized with chitosan molecules for greater stability and allow further functionalization to conjugate biomolecules to develop theranostic agents. Birma worked with Mn:ZnS QDs and coated them with chitosan bound to folic acid which has fluorescence intensity dependent on the concentration of Mn^2+^ doped.^38^

#### 4.2.3 Carbon Nanostructures

Several highly biocompatible nanostructures made out of carbon source are available with unique luminescent properties. Graphene dots of diameter 1.65 nm prepared through Microwave Assisted Hydrothermal method with glucose as the sole carbon source are very similar to the carbon dots but they have crystalline structure with auto passivated surface. Also, in the study conducted by Tang et al., deep ultraviolet emission is observed in graphene QDs attributed to the passivated surface instead of emission in the visible spectrum to negate the auto fluorescence.^39^ Their luminescent properties cannot be altered with size. Carbon dots with amorphous structure have attracted a great deal of attention in the recent years due to their biocompatibility compared to all sort of nanosized particles. Carbon dots can produce fluorescence in comparison to QDs or more enhanced version is achieved by functionalizing their surface with passivating agents like PEG 1500, L‐tyrosine,^40^ etc. Functionalizing carbon dots again with carbon dots, that is cross linking them, produces photochemical stabilized particles with high brightness for applications requiring high sensitivity.^41^ Xu et al. reported that, simply adding GdCl_3_ to the solution mixture containing a carbon source(citrate acid) and passivating agent (ethanediamine) create better MRI agents than DTPA linked Gd particles because of high loading, with its quantum yield efficiency unchanged.^42^ Li et al. worked with multiwalled carbon nanotubes (MWCNTs) of diameter 40–60 nm and showed that those tubes can be oxidized to introduce carboxylic groups on the surface to attach with Fe(III) ions and later link with SPIONs through solvothermal procedures.^43^ These MWCNTs now can be analyzed through MRI along with fluorescence, therefore giving better details about the in vivo biodistribution and clearance even 30 d after injection. In the experiment conducted by Prabhakar et al., oxidized graphene QDs capable of tuning fluorescent properties showed a greater stability, high cellular uptake and its fluorescence is slightly increased instead of being quenched through the functionalization of polyethyleneimine (PEI)‐PEG‐Folic acid on its surface.^44^ The PEI directly attached increases the electrostatic attraction and folic acid helps in targeting the cancer cells through overexpression of folate receptors. Wang et al. reported that Octa‐aminopropyl Polyhedral Oligomeric Silsesquioxane Hydrochloride Salt (OA‐POSS) acts as a passivating agent for the carbon dots.^45^ Here, the carboxylic group reacts with the amine group in the OA‐POSS. The CDs with 3.6 nm were soluble in water with 24% of quantum yield efficiency. Yang et al. produced CDs from proteins like Bovine Serum Albumin (BSA) with quantum yield efficiency of 34.8% and used for tracking the Transacting Activator of Transcription peptide uptake in HeLA cells by conjugating it with the NPs.^46^ Such a bright fluorescence is obtained with which analysis can be done at a single particle level.

### 4.3 Magnetic Resonance Imaging

**Figure 4:**
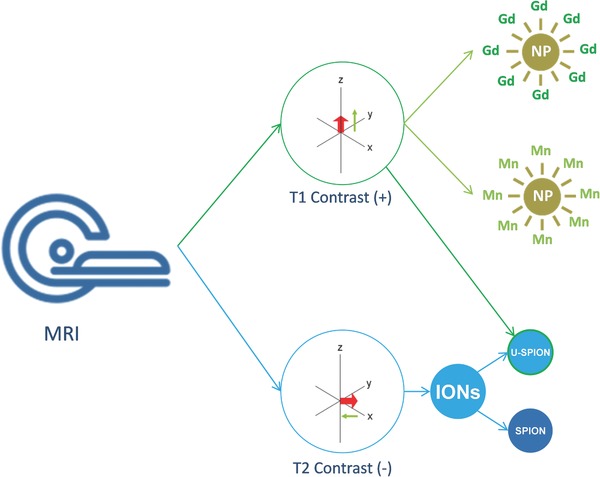
T1 and T2 type contrast in MR imaging. Gd(III) is usually preferred as positive contrast imaging agent. Prolonged positive contrast imaging can be done by functionalization of contrast agents to the nanoparticles. Iron oxide particles are T2 contrast agents and ultrasmall nanoparticles are dual contrast (T1/T2) agents.

NPs are used and replaced over other CAs because of the long blood circulation times useful for prognosis and it can be functionalized to improve its functions.^47^ In MRI, there are two types of CAs—negative and positive which are also termed as T2 weighted and T1 weighted, respectively. Magnetic NPs like IONPs are negative CAs with r2 relaxivity. But efficient contrast images can be captured by using T1 weighted compound like soluble gadolinium.^48^ **Figure** **4** shows the classification of MRI CAs. Modifying the surface with these CAs is more beneficial than core doping as the protons in the tissue has to interact with CAs to produce images with specific intensity.^49^ Highly biocompatible and biodegradable NPs are preferable for theranostic applications. Ratzinger et al.'s researched on surface functionalization of PLGA NPs with Gd^3+^, fixing it with ligands like DTPA or DOTA to introduce reactive groups which can covalently attach with Gd^3+^ and other molecules on the surface, preventing their release proves the particles transform as efficient MRI CAs.^6^, ^50^ Polymers like Polysilsesquioxane which are extremely biocompatible, easily available and its physiochemical properties can be changed very easily was surface functionalized with Gd(III) by Vivero‐Escoto et al. to make CAs.^51^ Also to reduce the signal to noise ratio, functionalization with PEG or molecules like anisamide which decreases the cytotoxicity reduces the interaction with the protons present in the water molecules. The signal produced is mainly due to the interaction with tissue. This way, the CA's efficiency and quality increases. In case of negative CAs, the ability to quench the MRI signal determines its efficiency.

Rosenberger et al. encapsulated magnetic NPs encapsulated with human serum albumin, which proved to act as efficient quenchers even after highly diluting the tracer.^52^ The human serum albumin provides the reactive functional groups on the surface to make them multifunctional. Mostly positive contrast images are preferred for analysis. Ultrasmall iron oxide particles are capable to act as positive CAs and therefore there is no need of conjugating Gd. Thus, there is more room to functionalize the NPs, therefore Luo et al., to make them multi‐functional and less cytotoxic, linked the NPs with Arg‐Gly‐Asp (RGD) peptide via PEG to target the integrin receptors to image angiography and glioma.^53^

PEG‐SPION are known to show increased relaxivity times, thus enhancing the image contrast in MRI. Also, they can act efficiently as dual CA for both T1 and T2 weighted in vitro MRI. However in vivo T1 weighted imaging with it cannot be done as aggregation occurs due to opsonization by immune cells.^21^ The improvement in signal contrast provided by PEGlyated SPION allow to diagnose diseases like atherosclerosis which was not possible before functionalization due to low signal enhancement and high toxicity concerns.^54^

MRI based nanorobotic systems based on NPs are in research and development for various diagnostic and therapeutic applications. Tabatabaei et al. worked on the development of nanorobotic carriers for delivery of drugs which used localized hyperthermia to disrupt BBB based on the fact that alternating magnetic fields excites the NP and causes elevation in temperature.^55^ Similarly, Martel et al. showed that magnetotactic bacteria can be loaded with therapeutic agents with real‐time navigation and control through MRI based feedback.^56^

### 4.4 Positron Emission Tomography

**Figure 5:**
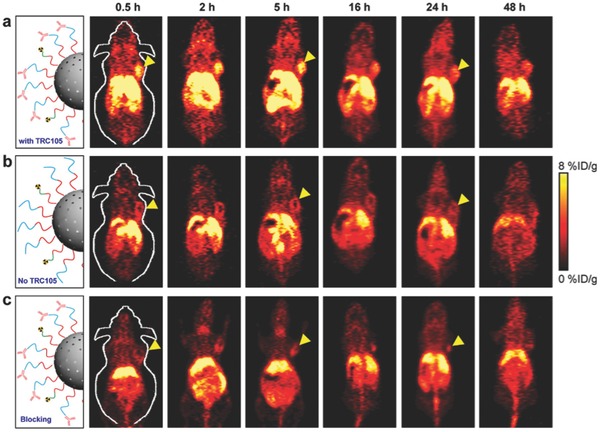
Serial coronal PET images of 4T1 tumor‐bearing mice at different time points postinjection of a) 64Cu‐NOTA‐mSiO2‐ PEG‐TRC105, b) 64Cu‐NOTA‐mSiO2‐PEG, or c) 64Cu‐NOTA‐mSiO2‐PEG‐TRC105 with a blocking dose of TRC105. Tumors were indicated by yellow arrowheads. Adapted with permission.^57^ Copyright 2013, American Chemical Society.

PET is a functional imaging modality which can analyze the metabolic processes and can detect diseases before structural modifications show up in the body. NPs are conjugated with radiotracers for enhanced and prolonged PET imaging. Chen et al. showed that Mesoporous Silica Particles (mSiO_2_) linked with TRC105 antibody which is specific to CD105 present in the murine breast cancer cells, which are also functionalized with thiol and PEG molecules for its stability reduce opsonization.^57^ Now when the mSiO2 NPs are attached with ^64^Cu radioisotope through the bond with NOTA (C_20_H_37_N_3_O_6_) chemical molecule functionalized mSiO2 NPs by treating with ^64^CuCl_2_, they become PET imaging agents as shown in **Figure** **5**.

Highly reproducible graphite oxide dots can be prepared with functionalizing ability. Yang et al. linked graphite oxide dot's surface to PEG molecules and IONPs to create MRI CAs or attached to iodine radioisotope I^125^ for a prolonged analysis through PET imaging.^58^ Biomimetic synthetic apatite NPs, also known as AP‐NPs were loaded with two radio isotopes by Sandhöfer et al., ^18^F directly inserted into the lattice and ^68^Ga attached by surface functionalization through NO_2_APBP, produced significant PET signals with stability of 90 min.^59^ NPs derived from the polyester 4‐(^18^F)fluorobenzyl‐2‐bromoacetamide ((^18^F)FBBA) were coupled to the block copolymers by Di Mauro et al. to image through PET and also to enable imaging through CT which can picture the fate of NPs.^60^

### 4.5 Computed Tomography

**Figure 6:**
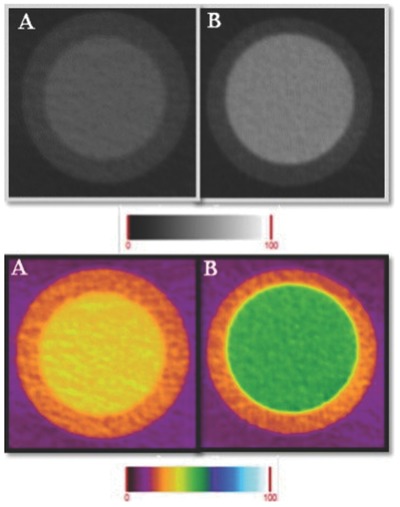
Micro‐CT grayscale (upper panel) and colored (lower panel) images of A) water and B) PEGylated Au NPs. Adapted with permission.^66^ Copyright 2015, Elsevier.

X rays can only give a 2D image, but CT, by utilizing X‐ray techniques can produce a 3D image. Liu et al. worked with BaYF5: Yb/Er NPs which allows CT imaging, as Ba and Yb ions which are doped into, can absorb X‐ray efficiently and also when excited by 908 nm, green and red upconversion emission was observed without any auto fluorescence.^61^ Through CT imaging modality, these dual modal probes were observed in the spleen for more than 2 h which can enable us to detect splenic diseases. Cole et al. reported that when gold NPs are linked with biphospahte, it can detect the microcalcifications composed of hydroxyl apatite which are beyond detection when nonfunctionalized particles were used.^62^, ^63^ Therefore, functionalization enhanced the image contrast by particular binding to Hydroxyapatite crystals. Gold nanorods can be used as CT imaging agents as well as gene carriers through functionalization of interested gene through poly(2‐(*N*,*N*‐dimethyl amino)ethyl methacrylate).^64^ Lalwani et al. incorporated manganese ions within the graphene sheets and functionalized with iodine in equimolar concentration to create dual probes, enabling MR imaging and PET.^65^ They also reported that imaging through these sheets showed a higher CT about 3200% greater at equimolar concentration of iodine and manganese. Silvestri et al. proved in their research that gold NPs conjugated with PEG molecules created directly by digestive ripening process followed by metal vapor process result in uniform and desired size CT CAs.^66^ PEGlyated Au NPs of 3.8 nm removes the concerns regarding toxicity and enables stable imaging for a prolonged time. As shown in **Figure** **6**, the attenuation in tube containing PEGylated Au NPs were found to be 8 times higher than the normal in vivo tissue. **Table** **1** enlists the significant molecules functionalized for specific imaging techniques and the effect of functionalization in imaging.

**Table 1:** Various molecules functionalized for specific imaging techniques and the effect of functionalization in imaging

Sl. No.	Functionalized molecule	Imaging technique	Effect of functionalization	Reference
1.	Specific fluorescent dyes	Fluorescence imaging, Confocal laser scanning microscopy	Enable imaging of various nanoparticles, NPs' interaction, dye's interaction with certain molecules or ions like Hg 2+ can detect their level	27
2.	Dyes and Quenchers	Fluorescence imaging	When the quenchers are cleaved as a result of interaction with the molecules attached to the quencher, fluorescence is seen.	98
3.	Methoxy peg attached to Quantum dots	Fluorescence imaging	Exposed to less toxicity while imaging due to reduced deposition of metal	22
4.	Porphyrin	Fluorescence imaging	Enables PDT to be carried out and allows TPE, therefore imaging at a greater depth is possible	92
5.	Aggregation induced dyes	Fluorescence imaging	Negates false positive results	36
6.	Zwitter ion ligands attached to quantum dots	Fluorescence imaging	Passivate the surface of particles thus enhancing the fluorescence	37
7.	PEG 1500, tyrosine attached to carbon nanostructures	Fluorescence imaging	Enhance the fluorescence by acting as passivating agents	40
8.	Carbon dots attached to another carbon dot	Fluorescence imaging	Photo chemically stabilized, bright fluorescence, high sensitivity	41
9.	Multiple layers of tryphtophan is attached to gold particles	Fluorescence imaging	Different number of layers yields different fluorescent properties due to change in lifetime of excited states, enabling imaging at a greater depth.	32
10.	Bi phosphate attached to Au particles	Computed Tomography	Enhances contrast, enabling the detection of Hydroxyl Apatite beyond the detection limit	62, 63
11.	Gd(III) ions	MRI(positive contrast)	Very high positive contrast imaging	6, 50
12.	Mn(II) ions	MRI	Positive contrast imaging but not that efficient as Gd(III)	99
13.	Ga 68	PET	Allows PET imaging, but has a shorter lifetime	87
14.	Iodine (125)	PET	This radioisotope has a greater life time	58

## 5 Functionalization to Enhance Theranostics

NP functionalization can enhance both, imaging and therapy simultaneously, thus development of theranostic nanoprobes for tumors is a major focus of various recent researches. The surface modification of NPs is known to provide targeted accumulation in tumors tissue due to Enhanced Permeability and Retention (EPR) effect.^67^ This helps in clear identification of area of interest in molecular imaging as well as targeted drug delivery. In comparison with normal tissue, the tumors have more permeable vasculature, poorly defined lymphatic system, and increased level of various other factors including vascular endothelial growth factor and basic fibroblast growth factor, and this differentiation assists in enhanced targeting.^68^ Xin et al. showed through in vivo fluorescence imaging that the Angiopep‐2 conjugated PEG–Polycaprolactone (PCL) NPs can selectively bypass the BBB and actively accumulates in glioma tissue due to EPR effect, in higher concentration than the nonfunctionalized NPs.^69^ This confirmed that conjugating NP with Angiopep‐2 induces glioma targeting capability through Lipoprotein Receptor‐related Protein (LRP)—mediated endocytosis in glioma cells.

In a study conducted by He et al., liposomes which have a lipid bilayer similar to the cell membrane was made multifunctional by loading in magnetic iron oxide NPs (MIONs) and a drug mitoxantrone which is effective against cancerous cells.^70^ This enabled the injected liposomes to be used as a CAs for MR imaging due to the MIONs and it can be directed against cancer cells. Further, for better targeting, MION was functionalized with gonadorelin which could interact with Luteinizing Hormone–Releasing Hormone (LHRH) and increase the uptake of drug in cells only with those with overexpressed LHRH receptors.

NPs alone were used for therapies instead of using carriers like liposomes through functionalization. Yang et al. showed that, iron sulfide (FeS) nanoplates when functionalized with PEG shows increased absorbance at near‐infrared (NIR) region and the NPs accumulated at the tumor sites, which allowed to conduct photothermal therapy.^71^ Also the FeS particles attached to PEG molecules showed higher r2 relaxivity than the Iron Oxide Particles (IONs) and thus prove to be a better diagnostic agent also. Zolata et al. worked with SPION, in which NPs were heavily functionalized to equip them with almost all functions possible.^72^ SPIONs surface was altered with 3‐Aminopropyltriethoxy Silane (APTES) and PEG on their surface to increase the half‐life and reduce toxicity. Then its surface was attached with the drug doxorubicin and biofunctionalized with antibody both used for cancer therapy. Also these particles were further labelled with indium‐111 to make them dual modality (SPECT/MR) probes. More specific drug delivery can happen through biofunctionalization. Aires et al. identified that targeted drug delivery to the CD44 positive cancerous cells can be done by functionalizing the magnetic iron oxide particles with antibodies(anti‐CD44) and gemcitabine derivatives used as anti‐cancer drugs.^73^ These particles were used as efficient theranostic agents.

As biodistribution of particles at nanoscale is dependent on many factors and undesired tissue uptake is well known issue, functionalization of NPs can also assist proper biodistribution^74^ and enhanced molecular level interaction.^75^

## 6 Functionalization to Enhance Targeting

**Figure 7:**
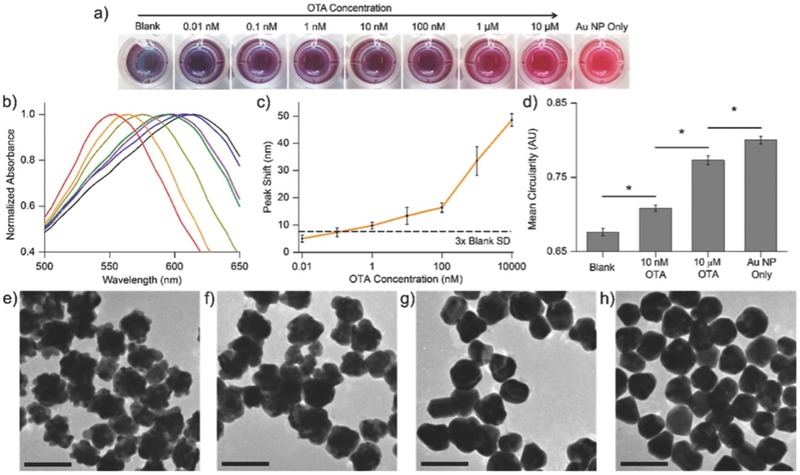
a) Colorimetric detection of OTA with the naked eye, whereby grown Au NPs changed from blue to red with an increasing concentration of OTA. “Au NP only” refers to Au NPs without OTA aptamer adsorption. b) UV–vis spectra of grown Au NPs corresponding to blank (black), 0.01 × 10^−9^ m (blue), 1 × 10^−9^ m (purple), 100 × 10^−9^ m (green), 1 × 10^−6^ m (gold), and 10 × 10^−6^ m (orange) OTA, and Au NPs without OTA aptamer adsorption (red). c) Peak shifts of various OTA concentrations measured with respect to the peak wavelength of the blank. Error bars indicate the SD of five independent experiments. d) Mean circularity of grown Au NPs, where circularity increased as grown Au NPs became more spherical morphologically. Error bars represent the standard error for *n* ≥ 250 NPs. Analysis of variance (ANOVA) using the Bonferroni test with pairwise comparison; ∗*P* < 0.01. TEM images of grown Au NPs for e) blank, f) 10 × 10^−9^ m OTA, g) 10 × 10^−6^ m OTA, and h) Au NPs without OTA aptamer adsorption. Scale bars = 100 nm. Adapted with permission.^82^ Copyright 2015, American Chemical Society.

Specific imaging of certain areas or cells to analyze a disease or tissue damage quantitatively is very important and that can be done only when the labelling is done with high specificity. In the case of malignant brain tumors, the glioblastoma cells (U87 cells) were confirmed with overexpressed Transferrin receptors. Ryoko Tsukamoto used QDs to label the U87 cells.^76^ QDs were surface functionalized with Transferrin and the labelling of U87 cells efficiency increased by 91.4 %. Alibolandi et al. in their research proved that CdTe QDs linked with AS1141‐aptamer, targeting the nucleolin, can efficiently enter the cancerous cells (U87).^77^ The QDs can also be functionalized with peptides like BSA and S. Hu et al. produced it in a single cost effective step using microfluidic chips.^78^ Borny et al. synthesized NPs out of Fe^3+^ and Fe^2+^ which were coated with dextran in a “hairy like” way and made biocompatible.^79^ Also the particles were detectable at very low concentration, which helps in early diagnostic approaches. To quantitatively measure the pancreatic beta cells which progressively decreases in those who suffer from diabetes mellitus, Ultrasmall Super Paramagnetic Iron Oxide Particles were functionalized by Burtea et al. with the peptide P88 which interacts with (FXYD2)Ya, a biomarker very specific to beta cells.^80^ Thus, these NPs helps to keep in check the count of cells. Also to study the nanoscale folding of DNA, Doane et al. functionalized quantum rods(QRs) with complementary ssDNA molecules which hybridized with the genome.^81^ The partial hybridization (protection) of short strands to ssDNA attached to QRs and by using mild Tm temperature (deprotection), better DNA coverage is possible through functionalization. As shown in **Figure** **7**, gold NPs linked with aptamers which target ochratoxin A(OTA) or 17β‐estradiol, that regulates various brain functions can be used to detect the concentration of these target substances through colorimetric assay.^82^ This assay has a high sensitivity and specificity because of the aptamers conjugated with the Au particles. It proves that the targeting efficiency increases through functionalization.

## 7 Functionalization to Enhance Multimodal Imaging

**Figure 8:**
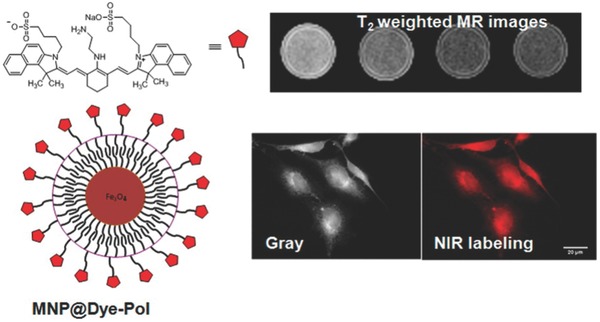
Amphiphilic polymer, poly‐(isobutylene‐alt‐maleic anhydride)‐functionalized near‐infrared (NIR) IR‐820 dye conjugated with iron oxide (Fe_3_O_4_) magnetic nanoparticles (MNPs) for optical and magnetic resonance (MR) imaging. Adapted with permission.^84^ Copyright 2013, American Chemical Society.

Multimodal imaging is a developing area where images from different equipment of the same scene is captured and superimposed to render multiple details such as the contrast, function, intensity, etc. Functionalization plays an important role in creating multimodal probes with ease. He et al. doped the UCNPs, having unique optical properties, with Gd^3+^ to create dual modal probes. Gd^3+^ is a positive CAs in MRI therefore the contrast is dependent on its concentration.^83^ These were further coated with PEG molecules, bi‐layered with PEI and its surface attached to DNA to create probes which could carry out gene transfection. Also it was observed that these UCNPs have greater R1 value than the DTPA Gd^3+^ solution. Yen et al., as shown in **Figure** **8**, functionalized the magnetic NPs, conjugated with polymers, with NIR dye(IR ‐820) and these particles now can be used for both optical and MR imaging which also exhibited less toxicity.^84^ The dye's Stokes shift can be tuned from 106 to 208 nm through functionalization.

Zhang et al. developed nanoprobe based on lanthanide for various image guided diagnosis with tunable MRI contrast and also for NIR activated Photodynamic Therapy (PDT).^85^ Xue et al. reported that SPIONs conjugated with 99mTc allows imaging through (SPECT).^86^ PET imaging is done to study the body functions through biochemical reactions. Therefore, before anatomical changes appear we can detect the formation of disease which is one great advantage over MR imaging. Pellico et al. identified that core doping iron oxide particles with 68 gallium, a radioisotope, will make dual modality‐PET/MRI probes.^87^ NIR Fluorescence Imaging (NIRF) is highly desirable as auto fluorescence by normal tissue is low at that excitation wavelength. QDs can absorb and emit in NIR region and does not lose its native properties even after heavy functionalization. Therefore, functionalization with various ligands each specific to a particular target can be made to diagnose multiple abnormalities. Hu et al. conjugated these QDs with 18 F radioisotope and therefore QDs can act as PET/NIRF probes to get a better insight.^88^ In multicomponent oxides like La1 xSrx MnO_3_ (LSMO), magnetic properties can be better tuned than the binary version‐iron oxides and when Kacˇenka et al. coated LSMO with the silica shell to which rhodamine molecules were linked, highly stabilized bimodal probes with increased florescence durability and improved magnetic properties were created through the silica shell covering.^89^ Zhang et al. created multi modal probes by loading micelles with Au NPs, an excellent CA for CT imaging, and AIE red dye, such that the features of the dye are undisturbed.^90^ CT/fluorescence probes with excellent penetration quality and high sensitivity are made possible by this way since Au is a strong quencher. Therefore, there is no need of injecting several probes specific to each modality.

## 8 Functionalization to Enhance Intraoperative Therapies

NPs largely used as probes can distinctly show the affected or diseased area with high contrast and good specificity. Simultaneously while imaging those areas if a therapy could be carried out, then the treatment can be done with high precision.

**Figure 9:**
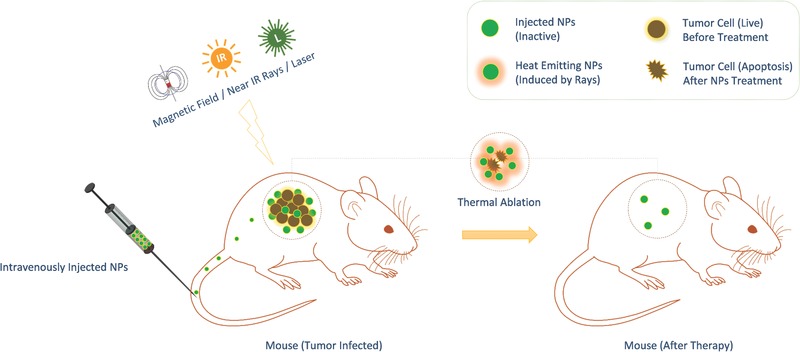
Image guided therapy—nanoparticles are injected intravenously into the mouse, targets the tumor cells, and get accumulated. When the mouse is exposed to radiation or specific waves, absorption at particular wavelengths causes the nanoparticles to heat up specifically to causes thermal ablation and clearance of tumor cells.

Immunotherapies for cancer can be done using NPs which help us to keep in track the immune cells useful during metastasis.^91^ When the energy absorbed is emitted as heat radiation then PDT is carried out to lyse the cells while imaging. For this, the NPs have to possess high specificity toward interested cells. Secret et al. showed that NPs attached to photosensitizers like porphyrin through covalent interactions can be used to carry out PDT and also through this attachment a Two Photon Excitation (TPE) can be done by transferring the energy after exciting the silica particle to the porphyrin.^92^ TPE allows us to do imaging at a greater depth and the silica particles loaded with mannose moieties help in targeting the interested cells. The amine groups linked to the YPO4: Eu NPs couple with carboxyl groups in the PEGylated iron oxide particles which make up the hybrid nanostructures. Barick et al. proved that the magnetic field induced by alternating current can cause self‐heating of these hybrid particles at highly specific absorption rates which enables us to control the therapy while imaging.^93^ Just the SPIONs have a good heating capacity of about 500 W g^−1^ Fe in alternating magnetic field of particular conditions of frequency. Kossatz et al. functionalized SPIONS with nucant multivalent pseudopeptide and doxorubicin through electrostatic interactions and reported that those SPIONs can be used to do magnetic hyperthermia therapy with enhanced cytotoxic effects toward the breast cancer cells.^94^ Jang et al. studied the thermal ablation of cancer cells with NIR laser light by irradiating nanographene oxide particles linked with two ligands, cyclic Arg‐Gly‐Asp (cRGD) and folic acid. They showed that in comparison with single ligand being attached, the combined effect of two ligands provides better targeting efficiency and also allows to clear the tumor growth at a large level due to the increased cellular uptake of these NPs.^95^ Liu et al. reported that, two dimensional MoS_2_ nano sheets can efficiently bind the iron oxide particles through sulfur and ^64^Cu can strongly be adsorbed onto its surface which allows photothermal therapy at greater accuracy through multimodal imaging (MRI, PET, Photoacoustic tomography).^96^ Here, the photothermal therapy was done by 808 nm laser light exposure given to mouse which can increase the temperature in the areas where the NPs are deposited, to 51 °C which can eradiate tumor. Recently NPs made of electrochemical polymers with a Negishi coupling at the end are found to be good candidates for use in photothermal therapy. Cantu et al. showed that suspension of these NPs increases by 30 °C by absorbing the laser light and converting to heat energy efficiently.^97^ **Figure** **9** explains the concept behind image guided therapy with thermal ablation.

## 9 Limitations and Perspectives

Even though various methods of functionalization and enhancing the molecular imaging are found and published in the discussed field, most of them are not reproducible exactly. Also the yield stated are very low which would require very large setup to commercialize the product. The functionalization processes require very different conditions to be prevailed for efficient surface modification. Also, the synthesis of NPs is an energy intensive process and obtaining uniform size is still not a reality in commercial aspects. Functionalization involves a lot of repeated processing of the NPs which further decreases the yield as in every chemical process we tend to lose the particles. Beside this, the clinical translations of these technologies are still not in proper practice, which is a major concern. The design considerations are extremely specific to facilitate various aspects of NP behavior in biological environment, and are composed of fundamental principles regulating clinical translation. The biocompatibility, efficient targeting ability, proper biodistribution and clearance of these nanoprobes are some of the key issues needed to be addressed with consistency in regulatory approval for their clinical applicability.

In future, more focus should be given to develop highly reproducible functionalization methods and incorporation of these nanoprobes to enhance molecular imaging, which can provide an approved clinical applicability. The functionalization of different molecules should be tried to be performed in a single step by having a common ligand/attaching chemical molecule. The size range of NPs should be still narrower for use as nanomedicine and nanoprobes. With newly developed efficient functionalization methods and robust design approaches, the upcoming researches are expected to well address the multidimensional regulatory aspects of clinical translation.

## 10 Conclusion

This review paper summarized the recent and current research going on in functionalizing the NPs and how these functional groups attached can modify the usage of NPs and accordingly enhance specific imaging modality. Conjugation of molecules equips them with multifunctional properties and open doors to multimodal imaging and various simultaneous therapies that can be combined while imaging through functionalization were discussed. Also functionalization done to the particle specific to the imaging techniques from in vitro to in vivo can improve the information that can be analyzed and ease of differentiated imaging through high contrast. The surface modification in addition to widening the application of NPs has also increased the efficacy of the particles by making the NPs biocompatible, increasing the stability of the particles and thus allowing prolonged imaging for tracking the interested cells over a period after injection to know the final fate of the NPs and its action. Though there are certain limitations in functionalizing NPs, controlled functionalization with desired molecules can greatly influence the transformation of nano medicine industry in future.

## References

[1] L. Chen , J. Li , M. Zhang , S. Cai , T. Zhang , C. Cai , Z. Chen , Med. Image Anal. 2015, 23, 1. 25910683 10.1016/j.media.2015.03.004

[2] P. Padmanabhan , A. Kumar , S. Kumar , R. K. Chaudhary , B. Gulyás , Acta Biomater. 2016, 41, 1. 27265153 10.1016/j.actbio.2016.06.003

[3] C. M. Goodman , C. D. McCusker , T. Yilmaz , V. M. Rotello , Bioconjugate Chem. 2004, 15, 897. 10.1021/bc049951i 15264879

[4] S. L. Montes‐Fonseca , E. Orrantia‐Borunda , A. Aguilar‐Elguezabal , C. González Horta , P. Talamás‐Rohana , B. Sánchez‐Ramírez , Nanomedicine 2012, 8, 853. 22033080 10.1016/j.nano.2011.10.002

[5] L. Bao , C. Liu , Z. L. Zhang , D. W. Pang , Adv. Mater. 2015, 27, 1663. 25589141 10.1002/adma.201405070

[6] F. Herranz , J. Pellico , J. Ruiz‐Cabello , SPIE Newsroom 2012, 1, 10.

[7] A. Aravind , P. Jeyamohan , R. Nair , S. Veeranarayanan , Y. Nagaoka , Y. Yoshida , T. Maekawa , D. S. Kumar , Biotechnol. Bioeng. 2012, 109, 2920. 22615073 10.1002/bit.24558

[8] R. Subbiah , M. Veerapandian , K. S. Yun , Curr. Med. Chem. 2010, 17, 4559. 21062250 10.2174/092986710794183024

[9] A. Fallis , Introduction to Biophotonics, John Wiley & Sons, Inc, Hoboken, NJ, USA 2013.

[10] H. Qu , S. Tong , K. Song , H. Ma , G. Bao , S. Pincus , W. Zhou , C. O'Connor , Langmuir 2013, 29, 10573. 23889037 10.1021/la4022867 PMC3905686

[11] H. S. Masatoshi , Y. Daisuke I. , Applications of Nanomaterials in Sensors and Diagnostics, Springer, Berlin 2013.

[12] D. Agudelo , L. Kreplak , H. A. Tajmir‐Riahi , Int. J. Biol. Macromol. 2016, 85, 150. 26723249 10.1016/j.ijbiomac.2015.12.057

[13] S. Li , Z. Gao , N. Shao , Talanta 2014, 129, 86. 25127568 10.1016/j.talanta.2014.04.062

[14] S. Svenson , R. I. Case , R. O. Cole , J. Hwang , S. R. Kabir , D. Lazarus , P. Lim Soo , P. S. Ng , C. Peters , P. Shum , B. Sweryda‐Krawiec , S. Tripathi , D. Van Der Poll , S. Eliasof , Mol. Pharmaceutics 2016, 13, 737. 10.1021/acs.molpharmaceut.5b00608 26835715

[15] S. K. uan Yen , D. P. Varma , W. M. ei Guo , V. H. B. Ho , V. Vijayaragavan , P. Padmanabhan , K. Bhakoo , S. T. amil Selvan , Chemistry 2015, 21, 3914. 25630810 10.1002/chem.201406388

[16] N. V Konduru , K. M. Murdaugh , A. Swami , R. J. Jimenez , T. C. Donaghey , P. Demokritou , J. D. Brain , R. M. Molina , Nanotoxicology 2016, 10, 720. 26581431 10.3109/17435390.2015.1113322 PMC4981177

[17] W. Ralph , R. D. Brian , R. Alnawaz , G. Sanjiv Sam , Molecular Imaging: Principles and Practice, PMPH‐USA, Shelton, CT 2009.

[18] H. Yu , M. Chen , P. M. Rice , S. X. Wang , R. L. White , S. Sun , Nano Lett. 2005, 5, 379. 15794629 10.1021/nl047955q

[19] J. A. Hachtel , S. Yu , A. R. Lupini , S. T. Pantelides , M. Gich , A. Laromaine , A. Roig , Faraday Discuss. 2016, 191, 215. 27419918 10.1039/c6fd00028b

[20] K. Avgoustakis , Curr. Drug Delivery 2004, 1, 321. 10.2174/1567201043334605 16305394

[21] L. Dai , Y. Liu , Z. Wang , F. Guo , D. Shi , B. Zhang , Mater. Sci. Eng. C 2014, 41, 161. 10.1016/j.msec.2014.04.041 24907749

[22] M. Ulusoy , R. Jonczyk , J. G. Walter , S. Springer , A. Lavrentieva , F. Stahl , M. Green , T. Scheper , Bioconjugate Chem. 2016, 27, 414. 10.1021/acs.bioconjchem.5b00491 26567697

[23] H. Kobayashi , M. W. Brechbiel , Adv. Drug Delivery Rev. 2005, 57, 2271. 10.1016/j.addr.2005.09.016 16290152

[24] H. S. Choi , W. Liu , P. Misra , E. Tanaka , J. P. Zimmer , B. Itty Ipe , M. G. Bawendi , J. V Frangioni , Nat. Biotechnol. 2007, 25, 1165. 17891134 10.1038/nbt1340 PMC2702539

[25] U. Kostiv , O. Janoušková , M. Šlouf , N. Kotov , H. Engstová , K. Smolková , P. Ježek , D. Horák , Nanoscale 2015, 18096. 26469980 10.1039/c5nr05572e

[26] A. Zarebkohan , F. Najafi , H. R. Moghimi , M. Hemmati , M. R. Deevband , B. Kazemi , Eur. J. Pharm. Sci. 2015, 78, 19. 26118442 10.1016/j.ejps.2015.06.024

[27] B. Gu , Y. Zhou , X. Zhang , X. Liu , Y. Zhang , R. Marks , H. Zhang , X. Liu , Q. Zhang , Nanoscale 2015, 8, 276. 10.1039/c5nr05286f 26607020

[28] M. Michalska , A. Florczak , H. Dams‐Kozlowska , J. Gapinski , S. Jurga , R. Schneider , Acta Biomater. 2016, 35, 293. 26850146 10.1016/j.actbio.2016.02.002

[29] E. Fanizza , R. M. Iacobazzi , V. Laquintana , G. Valente , G. Caliandro , M. Striccoli , A. Agostiano , A. Cutrignelli , A. Lopedota , M. L. Curri , M. Franco , N. Depalo , N. Denora , Nanoscale 2016, 8, 3350. 26763470 10.1039/c5nr08139d

[30] M. Martínez‐Calvo , K. N. Orange , R. B. P. Elmes , B. la Cour Poulsen , D. C. Williams , T. Gunnlaugsson , Nanoscale 2016, 8, 563. 26647086 10.1039/c5nr05598a

[31] M. Tamborini , E. Locatelli , M. Rasile , I. Monaco , S. Rodighiero , I. Corradini , M. Comes Franchini , L. Passoni , M. Matteoli , ACS Nano 2016, 10, 2509. 26745323 10.1021/acsnano.5b07375

[32] J. D. Pajović , R. Dojčilović , D. K. Božanić , S. Kaščáková , M. Réfrégiers , S. Dimitrijević‐Branković , V. V. Vodnik , A. R. Milosavljević , E. Piscopiello , A. S. Luyt , V. Djoković , Colloids Surf., B 2015, 135, 742. 10.1016/j.colsurfb.2015.08.050 26340364

[33] F. Natalio , A. Kashyap , S. Lorenz , H. Kerschbaumer , M. Dietzsch , M. N. Tahir , H. Duschner , S. Strand , D. Strand , W. Tremel , Nanoscale 2012, 4, 4680. 22735108 10.1039/c2nr30660c

[34] S. Wang , W. Xi , F. Cai , X. Zhao , Z. Xu , J. Qian , S. He , Theranostics 2015, 5, 251. 25553113 10.7150/thno.10396 PMC4279189

[35] J. L. Vivero‐Escoto , R. C. Huxford‐phillips , W. Lin , Chem. Soc. Rev. 2013, 41, 2673. 10.1039/c2cs15229k PMC3777230 22234515

[36] X. Wang , P. Song , L. Peng , A. Tong , Y. Xiang , ACS Appl. Mater. Interfaces 2016, 8, 609. 26653325 10.1021/acsami.5b09644

[37] M. Tasso , M. K. Singh , E. Giovanelli , A. Fragola , V. Loriette , M. Regairaz , F. Dautry , F. Treussart , Z. Lenkei , N. Lequeux , T. Pons , ACS Appl. Mater. Interfaces 2015, 7, 26904. 26551755 10.1021/acsami.5b09777

[38] I. B. Bwatanglang , F. Mohammad , N. A. Yusof , J. Abdullah , M. Z. Hussein , N. B. Alitheen , N. Abu , Int. J. Nanomed. 2016, 11, 413. 10.2147/IJN.S90198 PMC4730997 26858524

[39] L. Tang , R. Ji , X. Cao , J. Lin , H. Jiang , X. Li , K. S. Teng , C. M. Luk , S. Zeng , J. Hao , S. P. Lau , ACS Nano 2012, 6, 5102. 22559247 10.1021/nn300760g

[40] V. Gude , Beilstein J. Nanotechnol. 2014, 5, 1513. 25247134 10.3762/bjnano.5.164 PMC4168912

[41] P. Anilkumar , L. Cao , J. J. Yu , K. N. Tackett , P. Wang , M. J. Meziani , Y. P. Sun , Small 2013, 9, 545. 23413239 10.1002/smll.201202000

[42] Y. Xu , X. H. Jia , X. B. Yin , X. W. He , Y. K. Zhang , Anal. Chem. 2014, 86, 12122. 25383762 10.1021/ac503002c

[43] R. Li , R. Wu , L. Zhao , H. Qin , J. Wu , J. Zhang , R. Bao , H. Zou , Nanotechnology 2014, 25, 495102. 25409786 10.1088/0957-4484/25/49/495102

[44] N. Prabhakar , T. Näreoja , E. von Haartman , D. Sen Karaman , S. a. Burikov , T. a. Dolenko , T. Deguchi , V. Mamaeva , P. Hänninen , I. Vlasov , O. a. Shenderova , J. M. Rosenholm , Nanoscale 2015, 10410. 25998585 10.1039/c5nr01403d

[45] W.‐J. Wang , X. Hai , Q.‐X. Mao , M.‐L. Chen , J.‐H. Wang , ACS Appl. Mater. Interfaces 2015, 7, 16609. 26171887 10.1021/acsami.5b04172

[46] Q. Yang , L. Wei , X. Zheng , L. Xiao , Sci. Rep. 2015, 5, 17727. 26634992 10.1038/srep17727 PMC4669502

[47] M. M. El‐Hammadi , J. L. Arias , Expert Opin. Ther. Pat. 2015, 25, 691. 25800416 10.1517/13543776.2015.1028358

[48] C.‐T. Yang , P. Padmanabhan , B. Z. Gulyás , RSC Adv. 2016, 6, 60945.

[49] H. Du , J. Yu , D. Guo , W. Yang , J. Wang , B. Zhang , Langmuir 2016, 32, 1155. 26740341 10.1021/acs.langmuir.5b04186

[50] G. Ratzinger , P. Agrawal , W. Körner , J. Lonkai , H. M. H. F. Sanders , E. Terreno , M. Wirth , G. J. Strijkers , K. Nicolay , F. Gabor , Biomaterials 2010, 31, 8716. 20797782 10.1016/j.biomaterials.2010.07.095

[51] J. L. Vivero‐Escoto , W. J. Rieter , H. Lau , R. C. Huxford‐Phillips , W. Lin , Small 2013, 9, 3523. 23613450 10.1002/smll.201300198 PMC3804422

[52] I. Rosenberger , C. Schmithals , J. Vandooren , S. Bianchessi , P. Milani , E. Locatelli , L. L. Israel , F. Hübner , M. Matteoli , J. P. Lellouche , M. C. Franchini , L. Passoni , E. Scanziani , G. Opdenakker , A. Piiper , J. Kreuter , J. Controlled Release 2014, 194, 130. 10.1016/j.jconrel.2014.08.017 25173842

[53] Y. Luo , J. Yang , Y. Yan , J. Li , M. Shen , G. Zhang , S. Mignani , X. Shi , Nanoscale 2015, 7, 14538. 26260703 10.1039/c5nr04003e

[54] Y. C. Park , J. B. Smith , T. Pham , R. D. Whitaker , C. A. Sucato , J. A. Hamilton , E. Bartolak‐Suki , J. Y. Wong , Colloids Surf., B 2014, 119, 106. 10.1016/j.colsurfb.2014.04.027 PMC4108172 24877593

[55] S. N. Tabatabaei , S. Duchemin , H. Girouard , S. Martel , in Proc. IEEE Int. Conf. Robot. Autom. Saint Paul, Minnesota, USA 2012, 727. 10.1109/ICRA.2012.6225041 PMC3601978 23518572

[56] S. Martel , O. Felfoul , J.‐B. Mathieu , a. Chanu , S. Tamaz , M. Mohammadi , M. Mankiewicz , N. Tabatabaei , Int. J. Rob. Res. 2009, 28, 1169. 19890446 10.1177/0278364908104855 PMC2772087

[57] F. Chen , H. Hong , Y. Zhang , H. F. Valdovinos , S. Shi , G. S. Kwon , C. P. Theuer , T. E. Barnhart , W. Cai , ACS Nano 2013, 7, 9027. 24083623 10.1021/nn403617j PMC3834886

[58] K. Yang , L. Feng , H. Hong , W. Cai , Z. Liu , Nat. Protoc. 2013, 8, 2392. 24202553 10.1038/nprot.2013.146 PMC3878091

[59] B. Sandhöfer , M. Meckel , J. M. Delgado‐López , T. Patrício , A. Tampieri , F. Rösch , M. Iafisco , ACS Appl. Mater. Interfaces 2015, 7, 10623. 25915450 10.1021/acsami.5b02624

[60] P. P. Di Mauro , V. Gómez‐Vallejo , Z. Baz Maldonado , J. Llop Roig , S. Borrós , Bioconjugate Chem. 2015, 26, 582. 10.1021/acs.bioconjchem.5b00040 25710619

[61] H. Liu , W. Lu , H. Wang , L. Rao , Z. Yi , S. Zeng , J. Hao , Nanoscale 2013, 5, 6023. 23715609 10.1039/c3nr00999h

[62] L. E. Cole , T. Vargo‐gogola , R. K. Roeder , C. E. T. Al , ACS Nano 2014, 8, 7486. 24992365 10.1021/nn5027802

[63] L. E. Cole , T. Vargo‐Gogola , R. K. Roeder , ACS Nano 2015, 9, 8923. 26308767 10.1021/acsnano.5b02749

[64] P. Yan , N. Zhao , H. Hu , X. Lin , F. Liu , F. J. Xu , Acta Biomater. 2014, 10, 3786. 24814878 10.1016/j.actbio.2014.05.002

[65] G. Lalwani , J. L. Sundararaj , K. Schaefer , T. Button , B. Sitharaman , J. Mater. Chem. B 2014, 2, 3519. 10.1039/C4TB00326H PMC4079501 24999431

[66] A. Silvestri , L. Polito , G. Bellani , V. Zambelli , R. P. Jumde , R. Psaro , C. Evangelisti , J. Colloid Interface Sci. 2015, 439, 28. 25463172 10.1016/j.jcis.2014.10.025

[67] H. Maeda , Adv. Enzyme Regul. 2001, 41, 189. 11384745 10.1016/s0065-2571(00)00013-3

[68] S. D. Steichen , M. Caldorera‐Moore , N. A. Peppas , Eur. J. Pharm. Sci. 2013, 48, 416. 23262059 10.1016/j.ejps.2012.12.006 PMC3619023

[69] H. Xin , X. Jiang , J. Gu , X. Sha , L. Chen , K. Law , Y. Chen , X. Wang , Y. Jiang , X. Fang , Biomaterials 2011, 32, 4293. 21427009 10.1016/j.biomaterials.2011.02.044

[70] Y. He , L. Zhang , D. Zhu , C. Song , Int. J. Nanomed. 2014, 9, 4055. 10.2147/IJN.S61880 PMC4149452 25187709

[71] K. Yang , G. Yang , L. Chen , L. Cheng , L. Wang , C. Ge , Z. Liu , Biomaterials 2015, 38, 1. 25457978 10.1016/j.biomaterials.2014.10.052

[72] H. Zolata , F. Abbasi Davani , H. Afarideh , Nucl. Med. Biol. 2015, 42, 164. 25311750 10.1016/j.nucmedbio.2014.09.007

[73] A. Aires , S. M. Ocampo , B. M. Simões , M. Josefa Rodríguez , J. F. Cadenas , P. Couleaud , K. Spence , A. Latorre , R. Miranda , Á. Somoza , R. B. Clarke , J. L. Carrascosa , A. L. Cortajarena , Nanotechnology 2016, 27, 65103. 10.1088/0957-4484/27/6/065103 26754042

[74] G. Vignaroli , P. Calandro , C. Zamperini , F. Coniglio , G. Iovenitti , M. Tavanti , D. Colecchia , E. Dreassi , M. Valoti , S. Schenone , M. Chiariello , M. Botta , Sci. Rep. 2016, 6, 21509. 26898318 10.1038/srep21509 PMC4761914

[75] C. Yao , C. Wei , Z. Huang , Y. Lu , A. M. El‐Toni , D. Ju , X. Zhang , W. Wang , F. Zhang , ACS Appl. Mater. Interfaces 2016, 8, 6935. 26927957 10.1021/acsami.6b01085

[76] H. Y. Ryoko Tsukamoto , J. Cell Sci. Ther. 2013, 4, 150.

[77] M. Alibolandi , K. Abnous , M. Ramezani , H. Hosseinkhani , F. Hadizadeh , J. Fluoresc. 2014, 24, 1519. 25172439 10.1007/s10895-014-1437-5

[78] S. Hu , S. Zeng , B. Zhang , C. Yang , P. Song , T. J. Hang Danny , G. Lin , Y. Wang , T. Anderson , P. Coquet , L. Liu , X. Zhang , K.‐T. Yong , Analyst 2014, 139, 4681. 25054471 10.1039/c4an00773e

[79] R. Borny , T. Lechleitner , T. Schmiedinger , M. Hermann , R. Tessadri , G. Redhammer , J. Neumüller , D. Kerjaschki , G. Berzaczy , G. Erman , M. Popovic , J. Lammer , M. Funovics , Contrast Media Mol. Imaging 2015, 10, 18. 24753451 10.1002/cmmi.1595

[80] C. Burtea , S. Laurent , D. Crombez , S. Delcambre , C. Sermeus , I. Millard , S. Rorive , D. Flamez , M. C. Beckers , I. Salmon , L. Vander Elst , D. L. Eizirik , R. N. Muller , Contrast Media Mol. Imaging 2015, 10, 398. 25930968 10.1002/cmmi.1641

[81] T. L. Doane , R. Alam , M. M. Maye , Nanoscale 2015, 7, 2883. 25611367 10.1039/c4nr07662a

[82] J. H. Soh , Y. Lin , S. Rana , J. Y. Ying , M. M. Stevens , Anal. Chem. 2015, 87, 7644. 26197040 10.1021/acs.analchem.5b00875

[83] L. He , L. Feng , L. Cheng , Y. Liu , Z. Li , R. Peng , Y. Li , L. Guo , Z. Liu , ACS Appl. Mater. Interfaces 2013, 5, 10381. 24070392 10.1021/am403554x

[84] S. K. Yen , D. Janczewski , J. L. Lakshmi , S. Bin Dolmanan , S. Tripathy , V. H. B. Ho , V. Vijayaragavan , A. Hariharan , P. Padmanabhan , K. K. Bhakoo , T. Sudhaharan , S. Ahmed , Y. Zhang , S. Tamil Selvan , ACS Nano 2013, 7, 6796. 23869722 10.1021/nn401734t

[85] Y. Zhang , G. K. Das , V. Vijayaragavan , Q. C. Xu , P. Padmanabhan , K. K. Bhakoo , S. T. Selvan , T. T. Y. Tan , Nanoscale 2014, 6, 12609. 25185642 10.1039/c4nr01717j

[86] S. Xue , C. Zhang , Y. Yang , L. Zhang , D. Cheng , J. Zhang , H. Shi , Y. Zhang , J. Biomed. Nanotechnol. 2015, 11, 1027. 26353592 10.1166/jbn.2015.2023

[87] J. Pellico , J. Ruiz‐Cabello , M. Saiz‐Alía , G. del Rosario , S. Caja , M. Montoya , L. Fernández de Manuel , M. P. Morales , L. Gutiérrez , B. Galiana , J. A. Enríquez , F. Herranz , Contrast Media Mol. Imaging 2016, 11, 203. 26748837 10.1002/cmmi.1681

[88] K. Hu , H. Wang , G. Tang , T. Huang , X. Tang , X. Liang , S. Yao , D. Nie , J. Nucl. Med. 2015, 56, 1278. 26112023 10.2967/jnumed.115.158873

[89] M. Kačenka , O. Kaman , S. Kikerlová , B. Pavlů , Z. Jirák , D. Jirák , V. Herynek , J. Černý , F. Chaput , S. Laurent , I. Lukeš , J. Colloid Interface Sci. 2015, 447, 97. 25702866 10.1016/j.jcis.2015.01.071

[90] J. Zhang , C. Li , X. Zhang , S. Huo , S. Jin , F. F. An , X. Wang , X. Xue , C. I. Okeke , G. Duan , F. Guo , X. Zhang , J. Hao , P. C. Wang , J. Zhang , X. J. Liang , Biomaterials 2015, 42, 103. 25542798 10.1016/j.biomaterials.2014.11.053 PMC9518006

[91] J. Conniot , J. M. Silva , J. G. Fernandes , L. C. Silva , R. Gaspar , S. Brocchini , H. F. Florindo , T. S. Barata , Front. Chem. 2014, 2, 105. 25505783 10.3389/fchem.2014.00105 PMC4244808

[92] E. Secret , M. Maynadier , A. Gallud , A. Chaix , E. Bouffard , M. Gary‐Bobo , N. Marcotte , O. Mongin , K. El Cheikh , V. Hugues , M. Auffan , C. Frochot , A. Mor??re , P. Maillard , M. Blanchard‐Desce , M. J. Sailor , M. Garcia , J. O. Durand , F. Cunin , Adv. Mater. 2014, 26, 7643. 25323443 10.1002/adma.201403415

[93] K. C. Barick , A. Sharma , N. G. Shetake , R. S. Ningthoujam , R. K. Vatsa , P. D. Babu , B. N. Pandey , P. A. Hassan , Dalton Trans. 2015, 44, 14686. 26215789 10.1039/c5dt01522g

[94] S. Kossatz , J. Grandke , P. Couleaud , A. Latorre , A. Aires , K. Crosbie‐Staunton , R. Ludwig , H. Dähring , V. Ettelt , A. Lazaro‐Carrillo , M. Calero , M. Sader , J. Courty , Y. Volkov , A. Prina‐Mello , A. Villanueva , Á. Somoza , A. L. Cortajarena , R. Miranda , I. Hilger , Breast Cancer Res. 2015, 17, 66. 25968050 10.1186/s13058-015-0576-1 PMC4451751

[95] C. Jang , J. H. Lee , A. Sahu , G. Tae , Nanoscale 2015, 7, 18584. 26489965 10.1039/c5nr05067g

[96] T. Liu , S. Shi , C. Liang , S. Shen , L. Cheng , C. Wang , X. Song , S. Goel , T. E. Barnhart , W. Cai , Z. Liu , ACS Nano 2015, 9, 950. 25562533 10.1021/nn506757x PMC4351725

[97] T. Cantu , B. Rodier , Z. Iszard , A. Kilian , V. Pattani , K. Walsh , K. Weber , J. Tunnell , T. Betancourt , J. Irvin , J. Visualized Exp. 2016, 107, e53631. 10.3791/53631 PMC4781320 26780244

[98] S. Song , Y. Qin , Y. He , Q. Huang , C. Fan , H.‐Y. Chen , Chem. Soc. Rev. 2010, 39, 4234. 20871878 10.1039/c000682n

[99] D. Pan , A. Schmieder , S. Wickline , G. Lanza , Tetrahedron 2011, 67, 8431. 22043109 10.1016/j.tet.2011.07.076 PMC3203535

